# An implementation science approach to evaluating pathogen whole genome sequencing in public health

**DOI:** 10.1186/s13073-021-00934-7

**Published:** 2021-07-28

**Authors:** Angeline S. Ferdinand, Margaret Kelaher, Courtney R. Lane, Anders Gonçalves da Silva, Norelle L. Sherry, Susan A. Ballard, Patiyan Andersson, Tuyet Hoang, Justin T. Denholm, Marion Easton, Benjamin P. Howden, Deborah A. Williamson

**Affiliations:** 1 Microbiological Diagnostic Unit Public Health Laboratory, Department of Microbiology and Immunology, The University of Melbourne at The Peter Doherty Institute for Infection and Immunity, Melbourne, VIC Australia; 2 Centre for Health Policy, School of Population and Global Health, The University of Melbourne, Melbourne, Australia; 3 Victorian Tuberculosis Program, Melbourne Health, Melbourne, Australia; 4 Department of Microbiology and Immunology, The University of Melbourne at The Peter Doherty Institute for Infection and Immunity, Melbourne, Australia; 5 Victorian Health Department, Melbourne, Australia; 6 Department of Microbiology, Royal Melbourne Hospital, Melbourne, Australia

**Keywords:** Pathogen genomics, Evaluation, SARS-CoV-2, Listeria, Tuberculosis

## Abstract

**Background:**

Pathogen whole genome sequencing (WGS) is being incorporated into public health surveillance and disease control systems worldwide and has the potential to make significant contributions to infectious disease surveillance, outbreak investigation and infection prevention and control. However, to date, there are limited data regarding (i) the optimal models for integration of genomic data into epidemiological investigations and (ii) how to quantify and evaluate public health impacts resulting from genomic epidemiological investigations.

**Methods:**

We developed the Pathogen Genomics in Public HeAlth Surveillance Evaluation (PG-PHASE) Framework to guide examination of the use of WGS in public health surveillance and disease control. We illustrate the use of this framework with three pathogens as case studies: *Listeria monocytogenes*, *Mycobacterium tuberculosis* and SARS-CoV-2.

**Results:**

The framework utilises an adaptable whole-of-system approach towards understanding how interconnected elements in the public health application of pathogen genomics contribute to public health processes and outcomes. The three phases of the PG-PHASE Framework are designed to support understanding of WGS laboratory processes, analysis, reporting and data sharing, and how genomic data are utilised in public health practice across all stages, from the decision to send an isolate or sample for sequencing to the use of sequence data in public health surveillance, investigation and decision-making. Importantly, the phases can be used separately or in conjunction, depending on the need of the evaluator. Subsequent to conducting evaluation underpinned by the framework, avenues may be developed for strategic investment or interventions to improve utilisation of whole genome sequencing.

**Conclusions:**

Comprehensive evaluation is critical to support health departments, public health laboratories and other stakeholders to successfully incorporate microbial genomics into public health practice. The PG-PHASE Framework aims to assist public health laboratories, health departments and authorities who are either considering transitioning to whole genome sequencing or intending to assess the integration of WGS in public health practice, including the capacity to detect and respond to outbreaks and associated costs, challenges and facilitators in the utilisation of microbial genomics and public health impacts.

**Supplementary Information:**

The online version contains supplementary material available at 10.1186/s13073-021-00934-7.

## Background

Whole genome sequencing (WGS) is being incorporated into public health surveillance and disease control systems worldwide. The USA and the UK have both implemented national WGS services [1, 2], and in 2016, 26 European countries reported the use of WGS in routine public health practice [3]. The reduction in cost and advancement of portable sequencing technologies has continued to increase accessibility for low-resource settings and low- and middle-income countries [4–6].

Two major applications of pathogen WGS in disease control are (i) identifying and investigating outbreaks and (ii) genomic surveillance of pathogens of public health importance. WGS has higher discriminatory power than other genotyping methods and can divide clusters identified through other methods into more detailed groupings [7–9] and aid in mapping patterns of microbial relatedness across different human, animal and environmental samples [10]. A key advantage of WGS is the transferability of sequence data, which allows for rapid and interoperable national, international and cross-sectoral data sharing. This enhances the capacity of surveillance systems to quickly detect related cases where epidemiological links are difficult to identify. For example, epidemiological links may be difficult to identify in the case of geographically dispersed clusters, such as for food-borne illnesses caused by wide food dispersion, or temporally dispersed clusters, caused by pathogens with long incubation periods [11–14].

For laboratories, the direct costs of transitioning to WGS may include (i) the purchase of equipment and consumables; (ii) changes in laboratory workflows and methods, safety and training needs; and (iii) workforce remodelling [1]. In addition, the generation, analysis, visualisation and storage of genomic data require appropriate bioinformatics and data management infrastructure. Different analyses and reports may be produced based on the needs of diverse end users, and the reporting needs of end users may change according to circumstance, such as during an outbreak investigation. At present, standardised guidelines for the reporting of genomic data are limited [15–17], and end users may find it difficult to interpret the data being provided to them. Moreover, there may be end user variability in understanding the uses of genomics for public health, further impacting the utility of pathogen genomic data. Community expectations, including anticipated benefits and ethical concerns around privacy and stigma, also have implications for how pathogen genomic data are used, and the level of support and advocacy for its incorporation into public health systems [18–20].

To date, much of the literature on public health pathogen genomics comes from a research perspective. As such, it is often retrospective, illustrating ‘proof of concept’, and does not account for public health practice or outcomes [21–23]. Where there have been studies exploring the implications of public health utilisation of microbial genomics in relation to specific pathogens, the examination of public health practice has been undertaken in isolation from earlier steps such as sequencing and reporting practices, which have a profound impact on the utility of sequence data [24]. Further, the processes of workflow management, analysis and reporting have often been examined separately from the incorporation of genomic data into public health practice [1, 15]. To our knowledge, there are no whole-of-system evaluations of WGS in public health, nor any formal evaluation or implementation frameworks in this area. Yet, such an evaluation is critical for continued improvement of current genomic implementations, and to add to the evidence base available for other labs and jurisdictions currently embarking on this undertaking (Table 1). For these reasons, we developed the Pathogen Genomics in Public HeAlth Surveillance Evaluation (PG-PHASE) Framework, underpinned by the principles of implementation science, to examine the impact of WGS on public health. To illustrate application of the PG-PHASE Framework, we use three pathogens as case studies: *Listeria monocytogenes*, *Mycobacterium tuberculosis* and SARS-CoV-2.

**Table 1:** Potential benefits of evaluating WGS implementation in public health surveillance

Potential benefits	
Inform the development of appropriate guidelines and policies around the use of pathogen whole genome sequencing in public health practice	
Contribute to the body of evidence available regarding development of pathogen whole genome sequencing capacity in a considered, informed way	
Identify needed investment, infrastructure and training to successfully incorporate microbial genomics into public health practice	
Provide clarity around the expected outcomes of incorporating pathogen whole genome sequencing in public health and evidence of whether these outcomes are being realised	
Identify unexpected outcomes of pathogen whole genome sequencing in public health (either positive or negative) and take appropriate action as necessary	
Ensure the public health benefits of whole genome sequencing technology are equitably distributed across the population	
Identify and reduce inefficiencies or redundancies in the system	

## Methods

### Approach to designing the framework

The key objective of this study was to develop a framework for the evaluation of WGS implementation in public health. The framework development process consisted of three key stages: (i) a review of existing literature, (ii) a series of key stakeholder interviews with developers and end-users of pathogen genomic data and (iii) synthesis of information and design of the framework programme logic.

### Review of existing literature

A literature review was undertaken to identify studies incorporating (i) theorised advantages of WGS over traditional typing methods, (ii) experiences in transitioning to and utilising WGS in public health systems, (iii) evaluations of WGS utilisation in public health settings and (iv) frameworks for evaluation of surveillance systems. Specific search terms used are provided in Additional File 1, and a list of included studies are available in Additional File 2.

### Key stakeholder interviews

A series of interviews was undertaken with seventeen individuals involved in generating and using pathogen genomic data. Interviewees were selected in order to encompass a wide variety of roles related to the generation and utilisation of pathogen genomic data. Individuals included laboratory scientists, bioinformaticians, genomic epidemiologists, field epidemiologists, infectious diseases clinicians, clinical microbiologists and decision-makers involved in funding pathogen genomic implementation. Major themes covered in the interviews were:

- The transition to WGS and how practice differed before and after transition
- The analysis of genomic data
- Challenges in the transition to WGS and use of genomic data
- Differences in how WGS was used in relation to specific pathogens
- Anticipated and realised benefits of the transition to WGS (and who would accrue these benefits)
- What individuals thought was important for an evaluation framework to cover

Interviews were an iterative process; initial interviews were undertaken to draft the framework, which was then fed back to interviewees and refined again. Multiple interviews were undertaken with some interviewees. Data collection ended when there was sufficient understanding to construct the framework, and this understanding was confirmed through the iterative nature of the interview process.

### Designing and implementing the evaluation framework

Information from the literature review and stakeholder interviews was synthesised and used to design the framework. Two major approaches were used to underpin our framework: (i) the ‘*Framework for evaluating public health surveillance systems for early detection of outbreaks: Recommendations from the CDC Working Group*’ (the ‘CDC framework’) [25] and (ii) the ‘*Process evaluation of complex interventions: Medical Research Council guidance*’ (the ‘MRC framework’) [26]. The CDC framework outlines four categories to support evaluation of public health surveillance systems: system description, outbreak detection, experience, and conclusions and recommendations [25]. These four categories were used as the basis for conceptualising how the contribution of WGS to public health surveillance systems could be evaluated. The PG-PHASE Framework uses interviews with stakeholders, operational data and reports to develop a comprehensive description of how WGS data is produced and used, including system processes and understanding the perceived benefits from the perspective of both producers and users of data. The use of WGS data in outbreak detection is examined in the framework, focusing both on the sharing of genomic data to contribute to epidemiological investigations and on the perceived utility of the data shared. Ultimately, the framework is designed to identify strengths and weaknesses in WGS implementation and utilisation, and to subsequently produce recommendations to improve effectiveness and impact.

Where the CDC framework provides guidance on elements to be included for evaluations specific to surveillance systems, the MRC framework is focused more broadly on how to appropriately capture and assess the interdependencies and relationships inherent in complex interventions. The MRC framework was used to develop a framework that is sensitive to contextual factors while assessing not only impacts but elements of implementation and processes that generated impacts. Examination of laboratory processes, reporting mechanisms and the use of WGS data is situated within the needs of end users and the public health situation relative to the existing context. Processes and mechanisms are a key focus throughout, with an emphasis on why and how data is produced, shared and utilised—or not.

**Fig. 1:**
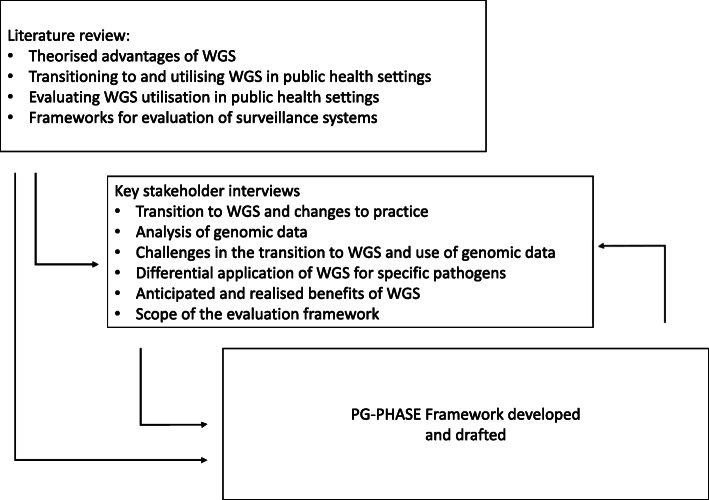
Development of the Pathogen Genomics in Public HeAlth Surveillance Evaluation (PG-PHASE) Framework

Finally, the phases for the framework were directly informed by the three phases of the total testing process in clinical laboratories (the pre-analytical, analytical and post-analytical phases). The final framework includes a range of data indicators and concepts (Additional File 3). The methods for development of the framework are illustrated in Fig. 1.

The evaluation framework can be broadly applied to many pathogens; however, components of the evaluation are likely to vary according to the specific pathogen and/or disease. We investigated preliminary ‘proof-of-concept’ of the framework by applying it theoretically to the evaluation of two major public health pathogens, namely *M. tuberculosis* and *L. monocytogenes.* Subsequently, to demonstrate ‘real-world’ utility of the framework, we applied the framework to contemporaneous implementation of SARS-CoV-2 sequencing in our setting.

## Results

The final evaluation framework is comprised of three phases, namely (i) the pre-analysis and analysis phase; (ii) the reporting and communication phase and (iii) the implementation phase. The associated outputs, outcomes and indicators for each phase are provided in Additional File 3 and are described further below.

### Phase 1: Pre-analysis and analysis

Given the relatively limited control the laboratory has regarding the pre-analytical phase (largely encompassing specimen selection, collection and transport), the pre-analytical and analytical phases are combined. This first phase focuses primarily on laboratory workflow in transitioning and undertaking WGS for the relevant pathogens. Initial ‘first-level’ data analysis is performed on individual sequences at this stage, including quality control, speciation, extraction of basic typing information and identifying the presence or absence of relevant loci/genes such as antimicrobial resistance (AMR) and virulence genes. Assessment encompasses (i) changes to workflow processes as ‘legacy’ laboratory methods are retired and pathogen characterisation is transitioned to WGS, (ii) the number of samples processed and analysed in a defined time period (efficiency) and (iii) costings, including costs for staff, instrumentation (including robotics) and reagents/consumables, and costs of data processing and storage. Decisions regarding which samples are selected for sequencing and sample processing and analysis times are also assessed as part of this phase of the evaluation. Elements of ‘future-proofing’ practices to ensure the future usability of isolates and sequence data, including adequate documentation of sample selection and how particular isolates are isolated, cultured and maintained, may also be explored [27]. Laboratory data, such as numbers of samples analysed, and purchasing data, such as reagent costs, may provide additional information. Interviews with laboratory staff may be useful to understand the impacts of workflow and other changes, such as change management, training staff in new processes and the adequacy of transition plans, as well as issues regarding sample transport and preparation. Interviews with both laboratory staff and end users may help define which isolates were selected for sequencing, why, and the role of laboratory and public health personnel in developing the sequencing strategy.

### Phase 2: Reporting and communication

Phase two examines reporting and communication processes from the dual perspectives of the laboratory and end-users. During this phase, ‘second-level’ analysis is undertaken, which relates first-level findings to additional metadata and extends analysis to a group of sequences (as distinct from an individual sequence) within the context of the request for analysis. Assessment in this phase encompasses the timeliness and utility of genomic data presented, and the structures in place to determine the most appropriate reporting formats and mechanisms, according to need. Reporting processes may vary and should be assessed according to (i) context and (ii) end user (e.g. epidemiologist, clinician). Evaluation of reporting processes may include the frequency of reports, level and type of information presented, visualisation of information, and adherence to information design principles [15, 28]. Included in this phase is an examination of the extent to which reporting mechanisms contribute to shared decision-making regarding sample selection, sequencing and analysis strategies between laboratory and public health personnel. Interviews with end users may include examination of intended use of genomic data and questions posed to inform analysis, satisfaction with communication and reporting processes, information retention, perceived utility of the information presented and level of understanding of information provided. Interviews with bioinformaticians and genomic epidemiologists can provide insight into the various ways sequence data is being used in addition to routine reporting (e.g. sequence data may be contributed to international databases, utilised in phylodynamic modelling or shared between public health agencies). Interviews may explore processes of data sharing, including data governance structures and legal or logistical barriers and facilitators. Through these interviews, areas for two-way learning may be identified, where end users are able to strengthen their understanding of the data provided, and bioinformaticians and genomic epidemiologists are better informed about what information is expected by end users and how it is intended to be used. Further, based on these interactions between those generating and those using genomic data, the format and contents of reports may be changed so that reports are better ‘fit for purpose.’

### Phase 3: Implementation in public health practice

This phase consists of two parts. The first is a qualitative examination of how WGS data is integrated into public health practice and used to complement or inform epidemiological investigations. Key informant interviews can assess the perspectives of stakeholders regarding the acceptability, usefulness and sustainability of pathogen genomics in public health. Key informants may include medical professionals such as physicians, microbiologists and epidemiologists, representatives of public health departments and hospital infection control units and other stakeholders (e.g. regulatory agencies, industry, community members). Interviews may explore possible applications and perceived utility or risks of pathogen genomics in infectious disease prevention and control, including the benefits and risks associated with WGS data storage and the ability to rapidly ‘mine’ large repositories of genomic data. Data may be collected on how the transition to WGS has affected public health decision-making, including confidence in making decisions based on the information provided, time to action and the types of decision-making enabled by genomic data. In addition to interviews, data collected may include documented changes to public health policy or the development of guidelines regarding the use of pathogen genomics in public health practice.

The second part of this evaluation phase is a quantitative examination of public health outcomes following implementation of pathogen genomics, with traditional laboratory processes (e.g. legacy typing methods) as a comparator. Relevant indicators may include epidemiological measures such as the number of identified cases; size, duration and number of identified clusters; the proportion of cases linked to clusters; and the proportion of cases/clusters traced to a contamination source. Although challenging to collect economic data, additional indicators may relate to financial costs such as resource allocation for epidemiological investigations and infection control investigations and actions [24, 29]; direct health care costs [30]; financial losses relating to food-borne disease outbreaks (e.g. food recalls); clean-up costs in relation to water and environmental outbreaks [29, 31, 32]; and costs associated with sick leave, both for employers in the form of reduced productivity and for employees as loss of income [30]. Data collection and analyses may vary significantly depending on the epidemiology of the pathogen under consideration, the type of data available and the context (i.e. routine surveillance or outbreak investigation). Indicators and data collection methods across the three phases of the evaluation framework are outlined in Additional File 4: Table S1.

### Evaluation case studies: *Listeria monocytogenes* and *Mycobacterium tuberculosis*

Listeriosis is a notifiable disease in many countries, including Australia and the USA [33–36]. WGS has emerged as a valuable tool for investigation of listeriosis outbreaks and is now routinely used for genomic surveillance in several countries [37, 38]. Previous research has shown that the higher discriminatory power of WGS can identify distinct nested clusters within groups of *L. monocytogenes* isolates that were otherwise indistinguishable using other typing methods [39] and has demonstrated utility in identifying contamination sources [40]. When there is data available from prior to the introduction of WGS, a pre/post study design can be used to examine data in defined time periods before (based on previously used typing methods) and after transitioning to WGS. Relevant variables to assess are detailed in Additional File 5: Table S2 and may include the number, size and geographical spread of identified clusters; the percent of isolates linked to a cluster; numbers of isolates/clusters traced to a common source; number of ‘solved’ isolates/clusters; and the time taken to identify and resolve outbreaks. Several of these outcomes have been assessed in genomic studies of other foodborne pathogens such as Shiga toxin-producing *Escherichia coli* [41, 42]. Jackson et al. examined listeriosis surveillance prior to and following integration of WGS, finding an increased number of clusters; identification of previously defined clusters that contained isolates that were not highly related; and the ability to link ‘unsolved’ cases to contamination sources [43]. Changes in costs relating to epidemiological investigation could also be analysed, given the utility of WGS in ruling out transmission links. The evaluation could additionally examine trends in food recalls due to *L. monocytogenes*, including the frequency and magnitude of recalls. A recent review identifies the application of WGS, including in industry, as key in increasing food safety and facilitating regulatory action to address listeria [44]. This approach allows for a comprehensive understanding of how the use of pathogen genomic data has affected the identification and characterisation of clusters across the surveillance system, as well as resulting effects on public health outcomes and use of public health resources.

Tuberculosis (TB) is the leading cause of death from a single infectious agent, with drug-resistant tuberculosis identified as a global health crisis [45]. The long incubation period and relatively high rates of asymptomatic and undiagnosed infection mean that it can be difficult to confirm transmission through epidemiological links alone. WGS has been shown to provide superior discrimination compared to other typing methods and may be more cost-effective [46, 47]. The use of WGS may therefore support more efficient and effective contact tracing, earlier and more appropriate treatment and the initiation of focused public health interventions [48, 49]. Suggested elements to include in an evaluation of TB WGS implementation are provided in Additional File 6: Table S3. Retrospective sequence data in combination with epidemiological data can be used to determine which TB cases may have been identified earlier, allowing for interventions to disrupt further transmissions. Estimates can then be made regarding the number of possible cases averted along with attendant costs to the health care system, including costs relating to epidemiological investigations that may not have been needed. Given the retrospective nature of this approach, it would be important to incorporate a strong understanding of how public health practice is informed by the use of pathogen genomic data, supported by phases 2 and 3 of the evaluation framework. As TB is a stigmatised illness, ethical considerations regarding the use of WGS, including issues around privacy; trust between individuals and communities affected by TB and public health agents and authorities; and community perception of the risks of WGS may also be explored as part of the evaluation [20]. Undertaking the evaluation in this way utilises a whole-of-system approach to draw links between how TB genomic data is used and eventual public health outcomes, enabling further refinement of pathogen genomics-informed public health practice.

### Evaluating the implementation of SARS-CoV-2 genomics

From the first instance of genomic sequencing of SARS-CoV-2 [50], WGS has been integrated into the global public health response to COVID-19 [13, 51–53]. To investigate the applicability of our framework, we applied it to the initial public health implementation of SARS-CoV-2 sequencing in Victoria, Australia. Additional file 7: Table S4 outlines the PG-PHASE Framework as applied to SARS-CoV-2 sequencing, with Table 2 outlining the data collection undertaken according to each phase of the evaluation.

**Table 2:** Data collection in application of the PG-PHASE Framework to SARS-CoV-2 sequencing

	Data collected in relation to each phase
**Phase 1**	• Number of sequenced cases • Number of total cases • Proportion of total cases sequenced • Turnaround time for all samples • Turnaround time for priority samples • Interviews with laboratory personnel • Interviews with end users
**Phase 2**	• Number of sequences uploaded to AusTrakka • Number of sequences uploaded to GISAID • Interviews with bioinformaticians • Interviews with genomic epidemiologists • Interviews with end users • Routine and ad-hoc reports • Observation of reporting meetings
**Phase 3 (part 1)**	• Interviews with end users • Media and publicly available inquiry records
**Phase 3 (part 2)**	• Estimated changes to health care costs due to cases averted • Estimated changes to costs of epidemiological investigation due to cases averted and use of WGS to identify transmission links • Estimated changes to costs of infection control interventions due to cases averted and use of WGS to identify transmission links

#### Phase 1: Pre-analytical and analytical evaluation of SARS-CoV-2 sequencing

The Microbiological Diagnostic Unit Public Health Laboratory (MDU PHL) located in Melbourne, Victoria, began routine sequencing of SARS-CoV-2 in January 2020. For phase 1, we collected both quantitative and qualitative data. Quantitative data were collected on the number of samples received and sequenced, turnaround times and quality control data. These data were analysed to enable understanding of sequencing efficiency and how this changed over the course of the pandemic. Qualitative data were used to contextualise this performance data. Seven laboratory staff and two staff involved in specimen transport and processing were interviewed. Semi-structured interviews were tailored to individuals’ roles and explored the processes of implementing WGS for an emerging pathogen, including changes to workflows, development of new analytical procedures and change management. These interviews provided a clearer understanding of factors that enabled the laboratory to respond effectively to an emerging pathogen and the issues involved in doing so. Interviews were undertaken at various stages throughout the pandemic. Later interviews were useful in understanding how earlier challenges were subsequently resolved.

#### Phase 2: Reporting and communication of SARS-CoV-2 genomic data

Interviews with laboratory personnel also explored reporting processes, including how bioinformaticians and genomic epidemiologists understood the data as being used in the public health response, possible or perceived deficiencies in how genomic data were understood by end users and the perceived appropriateness of data or analyses requested by end users. Interviews with bioinformaticians and genomic epidemiologists also covered mechanisms, facilitators and challenges in sharing SARS-CoV-2 sequence data, particularly across national and international boundaries. To complement interviews with laboratory personnel, eleven interviews were undertaken with end users of genomic data (Additional file 7: Table S4). Interviewees were asked how they received SARS-CoV-2 genomic data, what data were being requested and why, whether the data was provided in an appropriate and timely manner, whether reporting processes were responsive and appropriate to the intended use and to what extent they believed bioinformaticians and genomic epidemiologists understood the data needs of end users. Interviews explored perceived risks and challenges around data sharing and the use of genomic data in public health practice, including privacy issues. Throughout the pandemic response, the evaluator attended weekly reporting meetings that were held between the public health laboratory, Department of Health and others involved in the use of genomic data to inform the public health response. Where possible, regular and ad hoc reports were also collected, as well as emails requesting ad hoc analyses of SARS-CoV-2 genomic data. Data also included the number of sequences uploaded to public databases, which provides an indication of data sharing. The observation of the weekly reporting meeting and review of reports and ad hoc requests provided triangulation of the interview data for a complete picture of what genomic data was being requested and presented, and how producers and users of data worked together and communicated throughout the pandemic response. Collectively, these data were useful to identify possible misalignments between the perceptions of those generating and analysing genomic data and those who utilise the data to inform public health implementation.

#### Phase 3: Utilising SARS-CoV-2 genomic data in public health practice

Interviews with end users explored how genomic data was used in operationalising the public health response, both in community and hospital settings, and perceived barriers to its utilisation. Data in the public domain were used to understand how genomic data were involved in public health decision-making, including press releases from government officials referencing genomic data in explaining the rationale behind specific public health interventions (Additional file 7: Table S4). Within Victoria, genomic data played a key role in identifying the source of the second wave of infection as a breach in the state hotel quarantine system. In this case, evaluation data included the transcripts and final recommendations of a judicial enquiry into the Victorian hotel quarantine scheme [54]. Collectively, these data are valuable in identifying facilitators to the application of WGS in public health, addressing barriers to the utilisation of genomic data and refining appropriate public health decision-making practices informed by genomic data.

While these qualitative aspects of evaluation are essential, quantifying the impact of genomic sequencing is challenging for SARS-CoV-2 and other emerging pathogens, as there is no ‘counterfactual’ where WGS has not been used. Because of this, it is difficult to definitively attribute public health outcomes to the use of WGS, in comparison to an alternative situation where WGS is not available or utilised. Public health data from similar contexts where WGS has not been used, in conjunction with existing epidemiological data from the setting under consideration, could form the basis of mathematical models to estimate differences in case numbers and characterised outbreaks. While confounders would need to be accounted for in such a model, the aim would not be to arrive at absolute numbers, but rather relative results, such as the proportion of unidentified cases or the probability of being able to detect outbreaks of a certain size with and without the use of genomics [55]. In the absence of suitable comparative data, available epidemiological data may be examined to determine where identification of transmission events would have been uncertain, or where distinct transmission networks may have been merged without the use of genomic data.

## Discussion

Given the large investments that many laboratories and public health agencies have made in applying WGS to public health pathogen genomics [1, 56], it is essential to have a clear framework for evaluating the clinical, public health and economic impact (positive and negative) of WGS implementation and to derive the best value for money. Here, we present a framework for evaluating the use of WGS in public health surveillance and disease control across all stages, from the decision to send an isolate for sequencing, to the use of sequence data in public health surveillance, investigation and responses.

The benefits arising from transition to WGS and its subsequent use in public health surveillance are highly context dependent and rely on each part of the system working in conjunction with the others. Ideally, analysis, reporting and translation into practice work together as interconnected parts of an iterative process. Decisions regarding the selection and identification of isolates for sequencing may be informed by previously provided public health information, and WGS approaches need to be rapidly adaptable in order to respond to new and emerging pathogens and AMR. Analysis and reporting are dependent on the various needs of end users, which in turn are also influenced by the wider context. For this reason, a whole-of-system approach is necessary to understand how each step in the process of data generation, analysis and use are interconnected. The introduction of pathogen genomics into surveillance and disease control systems has had considerable impact on how laboratory and public health systems operate, with attendant uncertainty about the best approaches to: (i) facilitate the integration of genomic data into epidemiological investigations, (ii) define the necessary investment for pathogen genomics and (iii) evaluate public health impacts. The evaluation of public health programmes and interventions, including surveillance systems, is crucial to inform appropriate resource allocation, improve system responsiveness and ensure that programme goals are being met [57, 58]. The three phases of our evaluation framework are designed to support understanding of WGS laboratory processes, analysis, reporting and data sharing, and how genomic data are utilised in public health practice. Importantly, the phases can be used separately or in conjunction, depending on the need of the evaluator.

To test our framework, we applied it to SARS-CoV-2 sequencing in our setting and assessed the feasibility of collecting specific indicators that would enable a comprehensive evaluation. Phase 1 of the evaluation highlighted elements established before the pandemic that contributed to the laboratory being able to rapidly develop and implement protocols for sequencing and analysis of a new pathogen. Interviews with end users and documentation regarding public health decision-making provided an understanding of how genomic data informed (and continues to inform) public health practice. Data collection for this evaluation is ongoing, and the evidence arising from the final evaluation will provide guidance on appropriate investment for future pandemic preparedness planning, particularly for emerging pathogens. Moreover, the evaluation will help to develop a better understanding of the processes by which genomic data is effectively utilised in public health practice, thereby improving the impact of WGS and strengthening the use of genomics in public health surveillance systems.

## Conclusions

WGS is now a major part of public health surveillance and the control of infectious diseases, yet there are no defined ways of measuring the overall utility of this approach. This is a ‘first-in-field’ framework for the evaluation of whole genome sequencing (WGS) implementation in public health surveillance and outbreak investigation. The current evaluation framework is presented as a conceptual model, through a whole-of-system lens, to identify barriers and facilitators to the acceptable utilisation of WGS in public health throughout the process. Rigorous evaluation is critical for continued improvement in public health implementation of pathogen genomics and will increase clarity among stakeholders regarding expected outcomes and whether the aims of the programme are being achieved. This framework responds to a global increase in pathogen WGS in surveillance systems and a growing need for rigorous evaluation to support effective and efficient integration of pathogen WGS in public health. Ultimately, it is our hope and expectation that utilisation of the evaluation framework will support more effective and efficient integration of pathogen WGS in public health, leading to improved resource allocation, strengthened and more responsive surveillance systems and improved public health outcomes.

## Supplementary Information

**Additional file 1.** Literature review search terms and databases.

**Additional file 2.** Studies and reports included in the literature review.

**Additional file 3.** The evaluation framework: Key activities in utilising pathogen WGS in public health and associated outputs, outcomes and indicators.

**Additional file 4: Table S1.** Indicators and data collection methods.

**Additional file 5: Table S2.** Application of the evaluation framework for Listeria monocytogenes WGS.

**Additional file 6: Table S3.** Application of the evaluation framework for Mycobacterium tuberculosis WGS.

**Additional file 7: Table S4.** Application of the evaluation framework for SARS-CoV-2 WGS.

## Data Availability

Not applicable.
