# Supplementary material for: An implementation science approach to evaluating pathogen whole genome sequencing in public health
Source: Genome Med. 2021 Jul 28;13:121. doi: 10.1186/s13073-021-00934-7 (PMC8317677; doi:10.1186/s13073-021-00934-7)
Supplement: Supplementary file 1 — Additional file 1. Literature review search terms and databases. [file 13073_2021_934_MOESM1_ESM.docx]

**Additional file 1. Literature review search terms and databases**

**Literature review search terms**

- Whole genome sequencing OR WGS
- Next generation sequencing OR NGS
- Pathogen genomics OR microbial genomics
- Implementation
- Evaluation
- Public health
- Surveillance
- Salmonella
- Listeria
- Tuberculosis

**Databases searched**

- Web of Science
- PubMed
- Google
