# Supplementary material for: An implementation science approach to evaluating pathogen whole genome sequencing in public health
Source: Genome Med. 2021 Jul 28;13:121. doi: 10.1186/s13073-021-00934-7 (PMC8317677; doi:10.1186/s13073-021-00934-7)
Supplement: Supplementary file 2 — Additional file 2. Studies and reports included in the literature review. [file 13073_2021_934_MOESM2_ESM.docx]

**Additional file 2.** **Studies and reports included in the literature review**

- Australian Department of Health. National Framework for Communicable Disease Control. Canberra, Australia: Commonwealth of Australia, 2014.
- Black A, MacCannell DR, Sibley TR, Bedford T. Ten recommendations for supporting open pathogen genomic analysis in public health. Nature Medicine 2020.
- Brown E, Dessai U, McGarry S, Gerner-Smidt P. Use of whole-genome sequencing for food safety and public health in the United States. Foodborne Pathogens and Disease 2019; 16(7): 441-50.
- Bryant JM, Grogono DM, Greaves D, et al. Whole-genome sequencing to identify transmission of Mycobacterium abscessus between patients with cystic fibrosis: a retrospective cohort study. Lancet 2013; 381(9877): 1551-60.
- Buehler JW, Hopkins RS, Overhage JM, Sosin DM, Tong V. Framework for evaluating public health surveillance systems for early detection of outbreaks: Recommendations from the CDC Working Group: Centers for Disease Control and Prevention, 2004.
- Calba C, Goutard FL, Hoinville L, et al. Surveillance systems evaluation: A systematic review of the existing approaches. BMC public health 2015; 15: 448.
- Centers for Disease Control and Prevention. Framework for program evaluation in public health. Atlanta GA: US Department of Health and Human Services, 1999.
- Charre C, Ginevra C, Sabatier M, et al. Evaluation of NGS-based approaches for SARS-CoV-2 whole genome characterisation. Virus Evol 2020; 6(2): 8.
- Constantinides B, Chau KK, Quan TP, et al. Genomic surveillance of Escherichia coli and Klebsiella spp. in hospital sink drains and patients. Microb Genomics 2020; 6(7): 13.
- Crisan A, McKee G, Munzner T, Gardy JL. Evidence-based design and evaluation of a whole genome sequencing clinical report for the reference microbiology laboratory. PeerJ 2018; 6: 25.
- Department of Health. National Microbial Genomics Framework 2019 – 2022 Australian Government, 2019.
- Fong D, Otterstatter M, Taylor M, Galanis E. Analysis of enteric disease outbreak metrics, British Columbia Centre for Disease Control, 2005-2014. Can Commun Dis Rep 2017; 43(1): 1-6.
- Ford L, Carter GP, Wang Q, et al. Incorporating whole-genome sequencing into public health surveillance: Lessons from prospective sequencing of Salmonella Typhimurium in Australia. Foodborne Pathogens and Disease 2018; 15(3): 161-7.
- Ford L, Wang QN, Stafford R, et al. Seven Salmonella Typhimurium outbreaks in Australia linked by trace-back and whole genome sequencing. Foodborne Pathogens and Disease 2018; 15(5): 285-92.
- Gardy JL, Loman NJ. Towards a genomics-informed, real-time, global pathogen surveillance system. Nature Reviews Genetics 2018; 19(1): 9-20.
- Gilchrist CA, Turner SD, Riley MF, Petri WA, Jr., Hewlett EL. Whole-genome sequencing in outbreak analysis. Clinical microbiology reviews 2015; 28(3): 541-63.
- Grant K, Jenkins C, Arnold C, Green J, Zambon M. Implementing pathogen genomics: A case study: Public Health England, 2018.
- Greig DR, Schaefer U, Octavia S, et al. Evaluation of Whole-Genome Sequencing for Identification and Typing of Vibrio cholerae. J Clin Microbiol 2018; 56(11): 8.
- Gurjav U, Outhred AC, Jelfs P, et al. Whole genome sequencing demonstrates limited transmission within identified Mycobacterium tuberculosis clusters in New South Wales, Australia. PLoS One 2016; 11(10): 12.
- Harris SR, Cartwright EJP, Torok ME, et al. Whole-genome sequencing for analysis of an outbreak of meticillin-resistant Staphylococcus aureus: a descriptive study. Lancet Infect Dis 2013; 13(2): 130-6.
- Jackson C, Gardy JL, Shadiloo HC, Silva DS. Trust and the ethical challenges in the use of whole genome sequencing for tuberculosis surveillance: a qualitative study of stakeholder perspectives. BMC Medical Ethics 2019; 20(1): 43.
- Jagadeesan B, Baert L, Wiedmann M, Orsi RH. Comparative analysis of tools and approaches for source tracking Listeria monocytogenes in a food facility using whole-genome sequence data. Front Microbiol 2019; 10(947).
- Jajou R, van der Laan T, de Zwaan R, et al. WGS more accurately predicts susceptibility of Mycobacterium tuberculosis to first-line drugs than phenotypic testing. Journal of Antimicrobial Chemotherapy 2019; 74(9): 2605-16.
- Joensen KG, Scheutz F, Lund O, et al. Real-time whole-genome sequencing for routine typing, surveillance, and outbreak detection of verotoxigenic Escherichia coli. J Clin Microbiol 2014; 52(5): 1501-10.
- Köser CU, Ellington MJ, Cartwright EJP, et al. Routine use of microbial whole genome sequencing in diagnostic and public health microbiology. PLoS Pathog 2012; 8(8): e1002824-e.
- Kwong JC, Lane CR, Romanes F, et al. Translating genomics into practice for real-time surveillance and response to carbapenemase-producing Enterobacteriaceae: evidence from a complex multi-institutional KPC outbreak. PeerJ 2018; 6: e4210.
- Kwong JC, McCallum N, Sintchenko V, Howden BP. Whole genome sequencing in clinical and public health microbiology. Pathology 2015; 47(3): 199-210.
- Kwong JC, Mercoulia K, Tomita T, et al. Prospective Whole-Genome Sequencing enhances national surveillance of Listeria monocytogenes. J Clin Microbiol 2016; 54(2): 333-42.
- Leekitcharoenphon P, Nielsen EM, Kaas RS, Lund O, Aarestrup FM. Evaluation of Whole Genome Sequencing for Outbreak Detection of Salmonella enterica. PLoS One 2014; 9(2): 11.
- Leopold SR, Goering RV, Witten A, Harmsen D, Mellmann A. Bacterial whole-genome sequencing revisited: Portable, scalable, and standardized analysis for typing and detection of virulence and antibiotic resistance genes. J Clin Microbiol 2014; 52(7): 2365-70.
- McDermott PF, Tyson GH, Kabera C, et al. Whole-genome sequencing for detecting antimicrobial resistance in nontyphoidal Salmonella. Antimicrob Agents Chemother 2016; 60(9): 5515-20.
- McDonnell J, Dallman T, Atkin S, et al. Retrospective analysis of whole genome sequencing compared to prospective typing data in further informing the epidemiological investigation of an outbreak of Shigella sonnei in the UK. Epidemiol Infect 2013; 141(12): 2568-75.
- Moore GF, Audrey S, Barker M, et al. Process evaluation of complex interventions: Medical Research Council guidance. BMJ : British Medical Journal 2015; 350: h1258.
- Nielsen LR, Alban L, Ellis-Iversen J, Mintiens K, Sandberg M. Evaluating integrated surveillance of antimicrobial resistance: experiences from use of three evaluation tools. Clin Microbiol Infect 2020; 26(12): 1606-11.
- Pightling AW, Pettengill JB, Luo Y, Baugher JD, Rand H, Strain E. Interpreting whole-genome sequence analyses of foodborne bacteria for regulatory applications and outbreak investigations. Front Microbiol 2018; 9: 1482-.
- Popovich KJ, Snitkin ES. Whole genome sequencing-Implications for infection prevention and outbreak investigations. Curr Infect Dis Rep 2017; 19(4): 7.
- Rantsiou K, Kathariou S, Winkler A, et al. Next generation microbiological risk assessment: opportunities of whole genome sequencing (WGS) for foodborne pathogen surveillance, source tracking and risk assessment. Int J Food Microbiol 2018; 287: 3-9.
- Rantsiou K, Kathariou S, Winkler A, et al. Next generation microbiological risk assessment: opportunities of whole genome sequencing (WGS) for foodborne pathogen surveillance, source tracking and risk assessment. Int J Food Microbiol 2018; 287: 3-9.
- Revez J, Espinosa L, Albiger B, et al. Survey on the use of whole-genome sequencing for infectious diseases surveillance: Rapid expansion of European national capacities, 2015–2016. Front Public Health 2017; 5(347).
- Rumore J, Tschetter L, Kearney A, et al. Evaluation of whole-genome sequencing for outbreak detection of Verotoxigenic Escherichia coli O157:H7 from the Canadian perspective. BMC Genomics 2018; 19(1): 870.
- Scharff RL, Besser J, Sharp DJ, Jones TF, Peter G-S, Hedberg CW. An economic evaluation of PulseNet: A network for foodborne disease surveillance. American Journal of Preventive Medicine 2016; 50(5): S66-S73.
- Smolinski MS, Crawley AW, Olsen JM. Finding outbreaks faster. Health Secur 2017; 15(2): 215-20.
- van der Werf MJ, Kodmon C. Whole-genome sequencing as tool for investigating international tuberculosis outbreaks: A systematic review. Front Public Health 2019; 7: 9.
- World Health Organization. Joint external evaluation of IHR core capacities of Australia. Geneva, Switzerland: WHO, 2018.
- World Health Organization. Whole genome sequencing for foodborne disease surveillance: Landscape paper. Geneva, Switzerland: WHO, 2018.
- World Health Organization. Whole genome sequencing for foodborne disease surveillance: Landscape paper. Geneva, Switzerland: WHO, 2018.
- Wyres KL, Nguyen TNT, Lam MMC, et al. Genomic surveillance for hypervirulence and multi-drug resistance in invasive Klebsiella pneumoniae from South and Southeast Asia. Genome Med 2020; 12(1): 16.
