# Supplementary material for: An implementation science approach to evaluating pathogen whole genome sequencing in public health
Source: Genome Med. 2021 Jul 28;13:121. doi: 10.1186/s13073-021-00934-7 (PMC8317677; doi:10.1186/s13073-021-00934-7)
Supplement: Supplementary file 3 — Additional file 3. The evaluation framework: Key activities in utilising pathogen WGS in public health and associated outputs, outcomes and indicators. [file 13073_2021_934_MOESM3_ESM.docx]

**Additional file 3.** **The evaluation framework: Key activities in utilising pathogen WGS in public health and associated outputs, outcomes and indicators**

**Phase 1: Pre-analysis and analysis**

*Key activities (what is done at this stage)*

- Sample selection, collection and preparation
- Laboratory processes to conduct whole genome sequencing
- Production of sequence and typing data
- Quality control of sequence and typing data
- Analysis of sequence data

*Outputs (what are the results of these activities)*

- Appropriate samples received by laboratory
- Sample quality is adequate for processing and analysis
- Streamlined laboratory workflow processes
- Isolate sequence data obtained
- Basic information on sequence (type, composition and quality)

*Outcomes (what is achieved)*

- Increased efficiency of workflow processes
- Reduction in turnaround times
- Reduction in analysis costs
- Increased resolution of genotypic data

*Indicators (how do we know)*

- Levels of satisfaction with sample selection
- Levels of satisfaction with sample transport and quality
- Number of samples processed per week
- Number of samples analysed per week
- Diversity of samples processed per week
- Sample quality upon reception
- Sample processing times
- Sample analysis times
- Staff, equipment and reagent costs
- Levels of satisfaction with workflow processes
- Quality control outcomes
- Discriminatory power (ability to differentiate among unrelated isolates)
- Typeability (ability to provide an unambiguous result for each isolate analysed)

**Phase 2: Reporting and communication**

*Key activities (what is done at this stage)*

- Reporting mechanisms between end users and laboratory established
- Establishment of reporting requirements for phylogeny and typing data
- Preparation of routine reports in a timely manner as per established requirements
- Preparation of reports on an ad hoc basis in a timely manner as per established requirements
- Mechanisms established to facilitate data sharing across jurisdictions
- Mechanisms established to facilitate data sharing across sectors
- Establishment of appropriate data governance to support data sharing
- Standards are in place to ensure interoperability of genomic data management systems
- Standards are in place to ensure potability of genomic data and data systems

*Outputs (what are the results of these activities)*

- Efficient and effective communication between bioinformaticians, genomic epidemiologists and end users
- Routine reports for phylogeny and typing data provided as agreed
- Ad hoc reports for phylogeny and typing data provided as agreed
- Reporting mechanisms enable shared decision-making regarding sample selection and sequencing
- Genomic data routinely shared across jurisdictions to support surveillance, investigation and public health interventions
- Genomic data routinely shared across sectors to support surveillance, investigation and public health interventions
- Effective use of and contribution to genomic databases

*Outcomes (what is achieved)*

- Bioinformaticians have a good understanding of the needs of end users
- Genomic epidemiologists have a good understanding of the needs of end users
- End users have a good understanding of the possible uses and limitations of sequence data
- Information provided to end users is relevant and responsive to needs
- Information provided to end users in understandable and useable form
- Improved capacity to share genomic data across jurisdictions and sectors
- Improved capacity to identify and respond to geographically dispersed clusters
- Improved capacity to respond to clusters involving humans, animals and environmental strains

*Indicators (how do we know)*

- Agreements in place regarding reporting processes
- Agreements in place regarding data sharing and rights to access
- Satisfaction with agreements governing data sharing and rights to access
- Mechanisms in place to facilitate data archiving, tracking, tracing, and sharing
- Satisfaction with mechanisms in place to facilitate data archiving, tracking, tracing, and sharing
- Routine reports requested, issued and received
- Ad hoc reports requested, issued and received
- Retention of key information by end users
- Perceptions of end users regarding their own understanding of the uses and limitations of microbial genomics
- Perceptions of end users regarding bioinformaticians’ understanding of their needs
- Perceptions of end users regarding genomic epidemiologists’ understanding of their needs
- Perceptions of bioinformaticians regarding their own understanding of the needs of end users
- Perceptions of genomic epidemiologists regarding their own understanding of the needs of end users
- Perceptions of bioinformaticians regarding the use of diverse genomic data systems
- End users’ perception of the appropriateness of information received (i.e., quality, quantity, utility)
- Geographically dispersed clusters identified
- Clusters identified across human, animal and environmental samples

**Phase 3: Implementation in public health practice**

*Key activities (what is done at this stage)*

- Genomic data is integrated with epidemiological investigations
- Routine surveillance conducted across jurisdictions
- Routine surveillance conducted across sectors
- Coordinated investigations and actions undertaken to respond to outbreaks within and across jurisdictions and international borders
- Coordinated investigations and actions undertaken to respond to outbreaks across sectors

*Outputs (what are the results of these activities)*

- Genomic data is used to support public health decision-making
- Epidemiological links are confirmed or excluded based on genomic data
- Identification of transmission networks
- Cross-jurisdictional and internationally dispersed clusters routinely identified
- Clusters routinely identified across human, animal and environmental samples
- Source contamination accurately identified in a timely manner
- Food recalls are precise and timely

*Outcomes (what is achieved)*

- Increased confidence in public-health decision-making
- Public health policies and guidelines are informed by microbial genomics
- Public health interventions are appropriately tailored
- Improved understanding of transmission networks
- More precise allocation of investigative resources
- Reduction in time to identify outbreaks
- Reduction in time to respond to an identified outbreak
- Reduction in average size of clusters
- Improvements in identifying source contamination
- Improvements in linking cases to source contamination
- Reduced financial loss due to food recalls
- Reduction in health care costs
- Reduction in illness and mortality

*Indicators (how do we know)*

- End users’ perceptions of utilisation of genomic data in public health decision-making
- Presence of public health policies and guidelines informed by microbial genomics
- Perceptions of affected communities of public health actions and interventions
- Indirect consequences (e.g., nosocomial infections leading to ward lockdowns)
- Time lapse between identification of cluster and public health action
- Size of identified clusters
- Proportion of cases linked to identified clusters
- Capture of cases identified without epidemiological data
- Magnitude of food recalls
- Precision of food recalls
- Number of notifiable illnesses
- Health care costs due to notifiable illnesses
- Mortality due to notifiable illnesses
