# Supplementary material for: An implementation science approach to evaluating pathogen whole genome sequencing in public health
Source: Genome Med. 2021 Jul 28;13:121. doi: 10.1186/s13073-021-00934-7 (PMC8317677; doi:10.1186/s13073-021-00934-7)
Supplement: Supplementary file 4 — Additional file 4: Table S1. Indicators and data collection methods. [file 13073_2021_934_MOESM4_ESM.docx]

**Table S1. Indicators and data collection methods**

| **Data collection methods** | **Indicators** |
| --- | --- |
| Routine operational laboratory data | - Number of samples processed per week - Number of samples analysed per week - Sample processing times - Sample analysis times - Staff, equipment and reagent costs - Quality control outcomes - Discriminatory power - Typeability - Diversity of samples processed per week |
| Interviews with public health laboratory personnel | - Levels of satisfaction with workflow processes - Agreements in place regarding data sharing and rights to access - Satisfaction with agreements governing data sharing and rights to access - Mechanisms in place to facilitate data archiving, tracking, tracing, and sharing - Satisfaction with mechanisms in place to facilitate data archiving, tracking, tracing, and sharing - Perceptions of genomic epidemiologists regarding their own understanding of the needs of end users - Perceptions of bioinformaticians regarding their own understanding of the needs of end users - Perceptions of bioinformaticians regarding the use of diverse genomic data systems - Quality of information provided - Routine reports issued - Ad hoc reports issued |
| Interviews with end users | - Agreements in place regarding reporting processes - Routine reports requested, issued and received - Ad hoc reports requested, issued and received - End users’ perceptions of utilisation of genomic data in public health decision-making - Retention of key information by end users - Perceptions of end users regarding their own understanding of the uses and limitations of microbial genomics - Perceptions of end users regarding genomic epidemiologists’ understanding of their needs - Perceptions of end users regarding bioinformaticians’ understanding of their needs - End users’ perception of the appropriateness of information received (i.e., quality, quantity, utility) - Perceptions of affected communities of public health actions and interventions - Presence of public health policies and guidelines informed by microbial genomics - Indirect consequences (e.g., nosocomial infections leading to ward lockdowns) |
| Public health data (i.e., notifiable illnesses, public health surveillance data) | - Time lapse between identification of cluster and public health action - Geographically dispersed clusters identified - Clusters identified across human, animal and environmental samples - Size of identified clusters - Capture of cases identified without epidemiological data - Proportion of cases linked to identified clusters - Number of notifiable illnesses - Health care costs due to notifiable illnesses - Mortality due to notifiable illnesses |
| Publicly available food recall data | - Magnitude of food recalls - Precision of food recalls |
