# Supplementary material for: An implementation science approach to evaluating pathogen whole genome sequencing in public health
Source: Genome Med. 2021 Jul 28;13:121. doi: 10.1186/s13073-021-00934-7 (PMC8317677; doi:10.1186/s13073-021-00934-7)
Supplement: Supplementary file 5 — Additional file 5: Table S2. Application of the evaluation framework for Listeria monocytogenes WGS. [file 13073_2021_934_MOESM5_ESM.docx]

**Table S2: Application of the evaluation framework for *Listeria monocytogenes* WGS**

| **Phase of evaluation** | **Possible data collection and evaluation outcomes** |
| --- | --- |
| **Phase 1: Pre-analysis and analysis** | - Laboratory data (e.g. number of Listeria isolates received and typed; existing typing methods used for Listeria such as PFGE or MLST; cost of current typing; changes in turnaround times ) - Direct financial cost of WGS implementation in laboratory (i.e. wet lab and bioinformatic workflows) - Interviews with laboratory staff (inclusive of bioinformatic staff) to discuss current and proposed workflows (e.g. differences in specimen receipt, handling and processing; staffing impacts on new workflows) |
| **Phase 2: Reporting and communication** | - Interviews with end-users to discuss implementation and assessment of Listeria WGS deployment (e.g. availablity of public health data; discussions with food industry partners about possible impact of WGS) - Interviews with genomic epidemiologists to discuss interpretation and integration of data (e.g. visualisation of phylogenetic data; assistance to end-users with interpretation of genomic data; development of reports for end-users) - Interviews with bioinformaticians to discuss analytical approaches (e.g. interpretation of genomic data; facilitating availability of genomic data; supporting development of reports; visualisation of information) |
| **Phase 3: Implementation in public health practice** | Part 1 (qualitative evaluation)   - Interviews with end-users to discuss acceptability and useability of genomic data (e.g. public health units; food industry representatives; epidemiologists) - Public inquiries and documentation of provision of advice and evidence to government departments in outbreaks and legal cases   Part 2 (quantitative evaluation)   - Comparison of number of outbreaks ‘solved’ following WGS implementation (i.e. food or environmental source for outbreak identified) - Characterisation of listeriosis outbreaks (e.g.number, size and spread of clusters; number of food recalls) - Indirect costs and benefits of WGS implementation (e.g. food recalls; number of listerioisis cases averted; impact on manufacturing / processing facilities; costs of epidemiological investigation pre and post-WGS implementation) |
