# Supplementary material for: An implementation science approach to evaluating pathogen whole genome sequencing in public health
Source: Genome Med. 2021 Jul 28;13:121. doi: 10.1186/s13073-021-00934-7 (PMC8317677; doi:10.1186/s13073-021-00934-7)
Supplement: Supplementary file 6 — Additional file 6: Table S3. Application of the evaluation framework for Mycobacterium tuberculosis WGS. [file 13073_2021_934_MOESM6_ESM.docx]

**Table S3: Application of the evaluation framework for *Mycobacterium tuberculosis* WGS**

| **Phase of evaluation** | **Possible data collection and evaluation outcomes** |
| --- | --- |
| **Phase 1: Pre-analysis and analysis** | - Laboratory data (e.g. number of TB isolates received and typed pre-WGS; existing typing methods used for TB such as RFLP or MIRU; cost of current typing; changes in turnaround times; which isolates are routinely typed; time to generate TB resistance data) - Direct financial cost of WGS implementation in laboratory (i.e. wetlab and bioinformatic workflows) - Interviews with laboratory staff discussing laboratory workflows (e.g. specimen handling and processing; staffing impacts on new workflows; additional biosafety considerations for TB WGS) |
| **Phase 2: Reporting and communication** | - Interviews with end-users to discuss implementation and assessment of TB WGS (e.g. TB clinicians; TB epidemiologists; patient groups) - Interviews with bioinformaticians to discuss analytical approaches to TB WGS (e.g. detection of TB resistance mutations and correlation with phenotype; interpretation of genomic data; facilitating availability of genomic data; supporting development of reports; visualisation of information) - Interviews with genomic epidemiologists to discuss interpretation and integration of data (e.g. visualisation of phylogenetic data; assistance to end-users with interpretation of genomic data; development of reports for end-users and integration into public health practice) |
| **Phase 3: Implementation in public health practice** | Part 1 (qualitative evaluation)   - Interviews with end-users to discuss acceptability and useability of genomic data (e.g. public health units; epidemiologists; TB clinicians)   Part 2 (quantitative evaluation)   - Comparison of number of linked TB cases pre- and post- WGS implementation - Characterisation of TB outbreaks (e.g.number, size and spread of clusters; number of contacts linked to cluster) - Time to results pre-and post-WGS implementation (e.g. isolate identification; AMR results; time to cluster identification) - Indirect costs and benefits of WGS implementation (e.g. costs of epidemiological investigation pre and post-WGS implementation) |
