# Supplementary material for: An implementation science approach to evaluating pathogen whole genome sequencing in public health
Source: Genome Med. 2021 Jul 28;13:121. doi: 10.1186/s13073-021-00934-7 (PMC8317677; doi:10.1186/s13073-021-00934-7)
Supplement: Supplementary file 7 — Additional file 7: Table S4. Application of the evaluation framework for SARS-CoV-2 WGS. [file 13073_2021_934_MOESM7_ESM.docx]

**Table S4: Application of the evaluation framework for SARS-CoV-2 WGS**

| **Phase of evaluation** | **Possible data collection and evaluation outcomes** |
| --- | --- |
| **Phase 1: Pre-analysis and analysis** | - Laboratory data (e.g. number of SARS-CoV-2 samples received and sequenced; time to generate sequence data; quality control data) - Interviews with laboratory staff (e.g. changes to laboratory workflows; development of new analytical proedures; specimen handling and processing) |
| **Phase 2: Reporting and communication** | - Interviews with end-users (infectious disease clinicians; health department personnel) to discuss reporting and reception of SARS-CoV-2 genomic data; requested analyses; perceived risks and challenges of using SARS-CoV-2 genomic data in public health practice - Interviews with bioinformaticians and genomic epidemiologists to discuss mechanisms to share SARS-CoV-2 sequence data; understanding of applicability of data to public health response; perceived appropriateness of requests from end users; assistance to end-users with interpretation of genomic data; identification and interpretation of mutations with potential to lead to variants of concern |
| **Phase 3: Implementation in public health practice** | Part 1 (qualitative evaluation)   - Interviews with end-users to discuss acceptability and useability of genomic data (e.g. public health units; infectious disease clinicians) - Press releases from government officials referencing genomic data in explaining public health interventions - Transcripts and final recommendations of a judicial enquiry into the Victorian hotel quarantine scheme   Part 2 (quantitative evaluation)   - Identification of where transmission events would have been uncertain or distinct transmission networks merged without use of genomic data - Characterisation of COVID-19 outbreaks (e.g.number, size and spread of clusters; number of contacts linked to cluster) in settings where WGS has and has not been used - Direct financial cost of WGS implementation in laboratory - Indirect costs and benefits of WGS implementation (e.g. estimated costs of public health interventions such as lockdowns with and without WGS) |
